# COVID-19 and thyroid function: What do we know so far?

**DOI:** 10.3389/fendo.2022.1041676

**Published:** 2022-12-19

**Authors:** Camila Lüdke Rossetti, Juliana Cazarin, Fabio Hecht, Fabyan Esberard de Lima Beltrão, Andrea Cláudia Freitas Ferreira, Rodrigo Soares Fortunato, Helton Estrela Ramos, Denise Pires de Carvalho

**Affiliations:** ^1^ Institute of Biophysics Carlos Chagas Filho, Universidade Federal do Rio de Janeiro, Rio de Janeiro, Brazil; ^2^ Postgraduate Program in Nutritional Sciences, Department of Nutrition, Center for Health Sciences, Universidade Federal da Paraíba, João Pessoa, Brazil; ^3^ Campus Duque de Caxias Professor Geraldo Cidade, Universidade Federal do Rio de Janeiro, Rio de Janeiro, Brazil; ^4^ Department of Biorregulation, Health Sciences Institute, Universidade Federal da Bahia, Salvador, Brazil

**Keywords:** COVID-19, SARS-CoV-2, subacute thyroiditis, non-thyroidal illness syndrome, NTIS, hypothyroidism, hyperthyroidism

## Abstract

Coronavirus disease 2019 (COVID-19) was characterized as a pandemic in March, 2020 by the World Health Organization. COVID-19 is a respiratory syndrome that can progress to acute respiratory distress syndrome, multiorgan dysfunction, and eventually death. Despite being considered a respiratory disease, it is known that other organs and systems can be affected in COVID-19, including the thyroid gland. Thyroid gland, as well as hypothalamus and pituitary, which regulate the functioning of most endocrine glands, express angiotensin-converting enzyme 2 (ACE2), the main protein that functions as a receptor to which SARS-CoV-2 binds to enter host cells. In addition, thyroid gland is extremely sensitive to changes in body homeostasis and metabolism. Immune system cells are targets for thyroid hormones and T3 and T4 modulate specific immune responses, including cell-mediated immunity, natural killer cell activity, the antiviral action of interferon (IFN) and proliferation of T- and B-lymphocytes. However, studies show that patients with controlled hypothyroidism and hyperthyroidism do not have a higher prevalence of COVID-19, nor do they have a worse prognosis when infected with the virus. On the other hand, retrospective observational studies, prospective studies, and case reports published in the last two years reported abnormal thyroid function related to acute SARS-CoV-2 infection or even several weeks after its resolution. Indeed, a variety of thyroid disorders have been documented in COVID-19 patients, including non-thyroidal illness syndrome (NTIS), subacute thyroiditis and thyrotoxicosis. In addition, thyroid disease has already been reported as a consequence of the administration of vaccines against SARS-CoV-2. Overall, the data revealed that abnormal thyroid function may occur during and in the convalescence post-COVID condition phase. Although the cellular and molecular mechanisms are not completely understood, the evidence suggests that the “cytokine storm” is an important mediator in this context. Thus, future studies are needed to better investigate the pathophysiology of thyroid dysfunction induced by COVID-19 at both molecular and clinical levels.

## 1 Introduction

In December, 2019, a pneumonia of unknown origin emerged in Wuhan, China. On January, the virus responsible for the pneumonia was identified as a new coronavirus, later named severe acute respiratory syndrome coronavirus 2, SARS-CoV-2, and the disease was named coronavirus disease 2019 (COVID-19) (1). The genetic material of the virus was rapidly sequenced (2), and the transmission could not be prevented. The viruses rapidly spread across China and then all over the world, so on March 11^th^ 2020, World Health Organization (WHO) declared that COVID-19 reached pandemic levels. Since then, life on earth was totally affected by the pandemic, with changes in the way humans interact with each other, millions of deaths, ills and a great economic impact. On August 20^th^, 2022, according to WHO, the number of confirmed cases was higher than 595 million and confirmed deaths surpassed 6.4 million.

Most coronavirus strains that infect humans cause a mild respiratory disease; however, SARS-CoV-2 causes a serious illness, which can lead to severe respiratory syndrome and eventually to death. The most frequent symptoms include fever, dyspnea, sore throat, anosmia, dysgeusia, fatigue, and can progress to pneumonia, acute respiratory distress syndrome, and multiorgan dysfunction. SARS-CoV-2 genetic material is a positive-sense single-stranded RNA and the virus consists of a spherical particle, enveloped, with a diameter of approximately 120 nm (3). The origin of SARS-CoV-2 is controversial and the Huanan seafood wholesale market in Wuhan was suggested to be the place where the virus jumped to humans, with bat (*Rhinolophus affinis*) and pangolin (*Manis javanica*) being most probably the natural and intermediate hosts, respectively (4).

## 2 COVID-19 and Thyroid gland: General concepts

**Figure 1:**
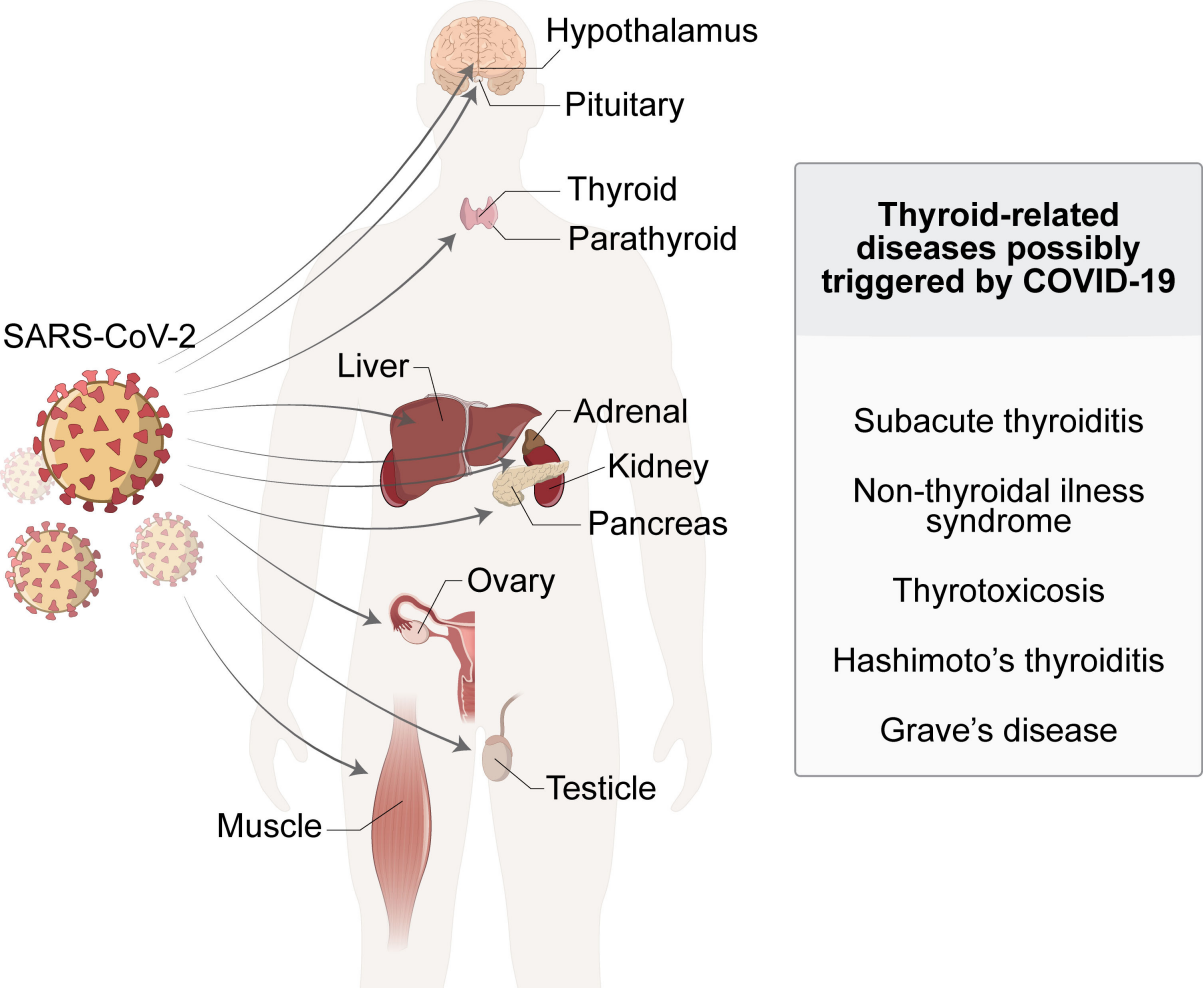
SARS-Cov-2 infection affects and damages a wide-ranging of human organs and systems. The manifestation of endocrine disorders, including thyroid-related pathologies, have been diagnosed in COVID-19 patients, suggesting a possible causal relationship between these conditions.

Despite being initially described as a respiratory disease, in the course of time it was observed that other organs and systems could be affected by COVID-19, such as cardiovascular system (5), central nervous system (6), kidney (7), liver (8), among others. Endocrine system is also affected by COVID-19, including pancreas, adrenal, testicle, reproductive tract, parathyroid gland and the thyroid gland (9–14) (**Figure 1**). Moreover, endocrine-metabolic disturbances, such as diabetes mellitus and obesity, are highly associated to severe illness (15, 16).

It is not surprising that the endocrine system can be affected by SARS-CoV-2 since both hypothalamus and pituitary, which regulate the functioning of most endocrine glands, express angiotensin-converting enzyme 2 (ACE2), the main protein to which SARS-CoV-2 binds to enter host cells (17, 18). ACE2 is a transmembrane protein with carboxypeptidase activity, which cleaves angiotensin I to angiotensin 1-9 and angiotensin II to angiotensin 1-7. The viral envelope contains a spike glycoprotein, which interacts with ACE2 with high specificity and affinity, which contributes to the high transmissibility and infectivity of SARS-CoV-2. Then, virus particle enters the cell by endocytosis or by fusion of the viral envelope with the cell membrane. The spike protein is not cleaved, thus the enzymatic activity of ACE2 is not relevant for the virus entry in host cell (19).

Besides hypothalamus and pituitary, thyroid gland also express ACE2 and may be directly affected by COVID-19 (20, 21). Thyroid gland is responsible for the production of thyroid hormones: the prohormone tetraiodothyronine (T_4_) and the active hormone triiodothyronine (T_3_). In fact, around 90% of the circulating T_3_ in humans is produced by the peripheral conversion of T_4_ to T_3_, by enzymes called deiodinases. T_3_ and some of its metabolites are the main regulators of basal metabolic rate, with effects on central nervous system, cardiovascular system, respiratory system, skeletal muscles, among others.

Thyroid hormones regulate to some extent the immune system (22). It has already been shown that one of the targets of thyroid hormones are immune system cells and that THs modulate specific immune responses, including cell-mediated immunity, natural killer cell activity, the antiviral action of interferon (IFN) and proliferation of T- and B-lymphocytes (22–24). In healthy subjects, there is a positive correlation between serum thyroid hormone levels and inflammatory markers, monocyte-activated IL-6 expression, the percentage of memory T cells, the quantity of natural killer T cells and the quantities of CD3^+^/CD4^+^/CD45RO^+^ memory T helper cells. On the other hand, serum thyroid hormones are negatively correlated with lymphocyte death and to the ratio of naïve: cytotoxic CD3^+^/CD8^+^/CD45RO^+^ memory T cells (25). These data suggest that THs stimulate the immune system to strongly react to infection.

Even though it has already been shown that hypothyroidism and hyperthyroidism have opposite effects on some parameters of the immune response, there is no evidence that patients with poorly controlled thyroid disorders are more susceptible to contract viral infections. However, considering the role of thyroid hormones on the immune system, it is plausible that patients with uncontrolled thyroid dysfunction may be at higher risk of complications due to these infections (26). Contrasting results have been reported for other immune functions, and so it is difficult to establish a clear correlation between immune function and hyper- or hypothyroid conditions (22). Overall, hypothyroidism tends to impair the activation of the immune system while hyperthyroidism results in the activation of the immune response (27).

Therefore, thyroid function could have an impact in the prognosis of COVID-19 and conversely COVID-19 could have an impact on thyroid function. In fact, abnormal thyroid function has been reported during SARS-CoV-2 infection or even several weeks after its resolution. Indeed, a variety of thyroid disorders have been documented in COVID-19 patients including non-thyroidal illness syndrome (NTIS), subacute thyroiditis (SAT), thyrotoxicosis and hypothyroidism in retrospective observational studies and case-reports (**Figure 1**). Although a cause-effect association between the infection and the onset of thyroid dysfunction has not yet been demonstrated from a mechanistic point-of-view, these reports raise the concern about whether thyroid function should or not receive a special attention in COVID-19 patients. Herein, we review important aspects of the relationship between COVID-19 and thyroid, including the interaction of thyroid hormones with immune system, the effect of infectious agents on the incidence of thyroid disorders and recent data regarding the relationship between COVID-19 and thyroid.

## 3 Pre-existing thyroid dysfunction and COVID-19

### 3.1 Hypothyroidism

Hypothyroidism is the insufficient production of thyroid hormones. This disease can be congenital, due to mutations in proteins that are essential for thyroid hormones synthesis pathway and defects in thyroid gland formation, or even the absence of the gland. Hypothyroidism can also be acquired, due to iodide deficiency, tumors or infections in the thyroid gland or in the pituitary, and autoimmunity. Hashimoto’s thyroiditis is an idiopathic thyroid atrophy due to a chronic autoimmune inflammatory reaction, being the most common form of hypothyroidism in humans (28). Hashimoto’s autoimmune thyroid disease is characterized by the production of autoantibodies against thyroglobulin (Tg-Ab) and thyroperoxidase (Tpo-Ab) that are essential for hormonal synthesis. Hypothyroid patients have lower metabolic rate, decreased thermogenesis, bradycardia, lethargy and drowsiness (29).

Animal models have shown that the experimental induction of hypothyroidism leads to an involution of the spleen and lymph nodes as well as a decrease in the humoral and cell-mediated immune response (30, 31). Hypothyroidism induced by chronic restraint stress seems to be related to the reduction of T-cell lymphoproliferative response, since T4 replacement reversed it. Besides, in these chronic stress mice bearing tumors, T4 reversed the alteration of lymphoma growth, interleukin-2 production and specific cytotoxic response against tumor cells (32). Furthermore, B-lymphocytes can also be regulated by thyroid hormones. In mouse strains deficient in the production of anterior pituitary-derived hormones, and consequently secondary hypothyroid, the frequency and absolute number of pro-B- and B-lymphocytes are lower, showing that THs can regulate the proliferative potential of T- and B-lymphocytes (33–35). Clinically, patients with severe hypothyroidism due to autoimmune thyroiditis experience a dramatic decrease in lymphocyte function, which is restored when T4 is normalized by exogenous hormone administration (36).

Considering that hypothyroidism leads to immune system dysfunctions, and that ACE2 is expressed in thyroid gland, one could speculate that hypothyroidism might impact the outcomes in COVID-19 patients. A retrospective study conducted in the New York City health system evaluated a cohort of 3703 COVID-19 patients, of which 251 patients (6.8%) had pre-existing hypothyroidism. The authors found that hypothyroidism was not associated with increased risk of hospitalization or an increased risk of mechanical ventilation or death (37). Other studies have also shown that the prevalence of hypothyroidism appears similar in COVID-19 patients compared to the general population, which indicates that hypothyroidism does not increase the chance of COVID-19 infection, and also that hypothyroidism is not associated with a greater COVID-19 death risk (38, 39). Despite this, previous studies show that, although well-managed hypothyroidism is not associated with increased infection risk, poorly controlled hypothyroidism may increase the susceptibility to infections (36, 40). Therefore, it is important that patients with thyroid disorders maintain their treatment during the COVID-19 pandemic.

### 3.2 Hyperthyroidism

Hyperthyroidism is characterized by higher levels of circulating thyroid hormone. It is mostly an acquired condition, which is most frequently caused by Graves’ disease, toxic multinodular goiter or toxic adenoma (41). Graves’ disease is an autoimmune disorder, in which thyroid-stimulating antibodies activate the thyroid-stimulating hormone (TSH) receptors, triggering increased thyroid hormone synthesis. The clinical condition observed in Graves’ disease is thyrotoxicosis, resulting from excessive amounts of thyroid hormones in the tissues and blood. In those patients, whole-body metabolism is activated leading to body weight loss, sweating, heat intolerance, increased heart rate, overactive bowel movement, tremor, nervousness, and exophthalmos (42).

Hyperthyroidism is associated with unbalanced immune responses, including abnormal antibody production (either increased or decreased) (43), increased migration of polymorphonuclear leukocytes (44), increased lymphocyte proliferation (45) and increased macrophages reactive oxygen species (ROS) production (27, 46). Compared to healthy controls, hyperthyroid patients present higher levels of serum immunoglobulins M and G (IgM, IgG) and higher levels of p65 and p-IκBα in B-lymphocytes, which are indicators of NF-κB activation. In addition, these patients have higher serum oxidative stress levels (47). These results suggest that hyperthyroidism increases ROS production activating the NF-κB pathway that, in turn, enhances the production of Ig’s by B-lymphocyte.

Due to this hyper-responsiveness of the immune system during hyperthyroidism, it is plausible that uncontrolled hyperthyroid patients, especially with thyrotoxicosis, may be at higher risk of complications from any infection (42). On the other hand, angiotensin converting enzyme activity and the counter-regulatory components of the RAS (renin-angiotensin system) is increased in patients with hyperthyroidism (48, 49). A study conducted in China shows that COVID-19 patients with thyroid disease had a significantly higher fatality rate (20% vs 0%) and they were more likely to stay in the hospital for more than 28 days than were those without thyroid disease (80% vs 56.52%) (50). However, the authors defined thyroid disease as an abnormal thyroid function test result, and included patients with overt thyrotoxicosis, overt hypothyroidism, subclinical hypothyroidism, subclinical hyperthyroidism, and euthyroid sick syndrome. Therefore, it is not possible to estimate the contribution of each thyroid disease to a worse prognosis of COVID-19.

In general, previously published data show that patients with controlled hyperthyroidism are not considered to be at higher risk of contracting COVID-19 (26, 51, 52), but there are two exceptions. It is known that patients taking antithyroid drugs present higher risk of developing neutropenia or agranulocytosis, which occurs in 0.2-0.5% of patients taking these medications (42, 53). Neutropenia is associated with increased risk of infections. Therefore, patients with neutropenia caused by the administration of antithyroid drugs may be more prone to complications during COVID-19 infection due to reduced immune response. Likewise, patients with Graves’ ophthalmopathy who are undergoing immunosuppressive agents, like glucocorticoids, may also be considered to be more vulnerable to COVID-19 infection (26, 51). Additionally, since thyroid hormones regulate vascular tonus and multi-organ dysfunction is associated to hypoxia, T3 treatment has been suggested to be potentially useful in the treatment of severe COVID-19 (54).

## 4 Thyroid dysfunction during COVID-19

### 4.1 Non-thyroidal illness syndrome (NTIS) and COVID-19

The first reports of abnormal serum thyroid hormone concentrations after severe illness or starvation in patients with no history of thyroid disease has been made nearly 60 years ago (55, 56). In mild-to-moderate illness, the most typical laboratory finding is a reduction in serum T_3_ and, remarkably, no concomitant increase in TSH. Accordingly, this condition has been named “low T_3_ syndrome”, “euthyroid sick syndrome” or “non-thyroidal illness syndrome”. Elevated reverse T_3_ (rT_3_) has long been considered another hallmark of NTIS, but now some authors argue that its levels may be normal or even reduced in some patients with NTIS (57). Reduced levels of rT3 have already been observed in AIDS patients that present NTIS, and this phenomenon has also been observed in COVID-19 and NTIS patients (58–60). Additionally, a decline in T_4_ and TSH may also be observed in critically ill patients. Thus, NTIS is a complex condition with no unique phenotype, which greatly depends on disease severity. Pro-inflammatory cytokines, like interleukin-6 and IL-1β, are recognized as major players in the pathogenesis of NTIS since the 90’s, when an inverse correlation between serum T_3_ and IL-6 in hospitalized patients was first observed (61). Moreover, the chronic treatment of rodents with these cytokines recapitulated several hallmarks of human NTIS, including low T_3_, T_4_ and hypothalamic TRH mRNA expression (62, 63).

**Figure 2:**
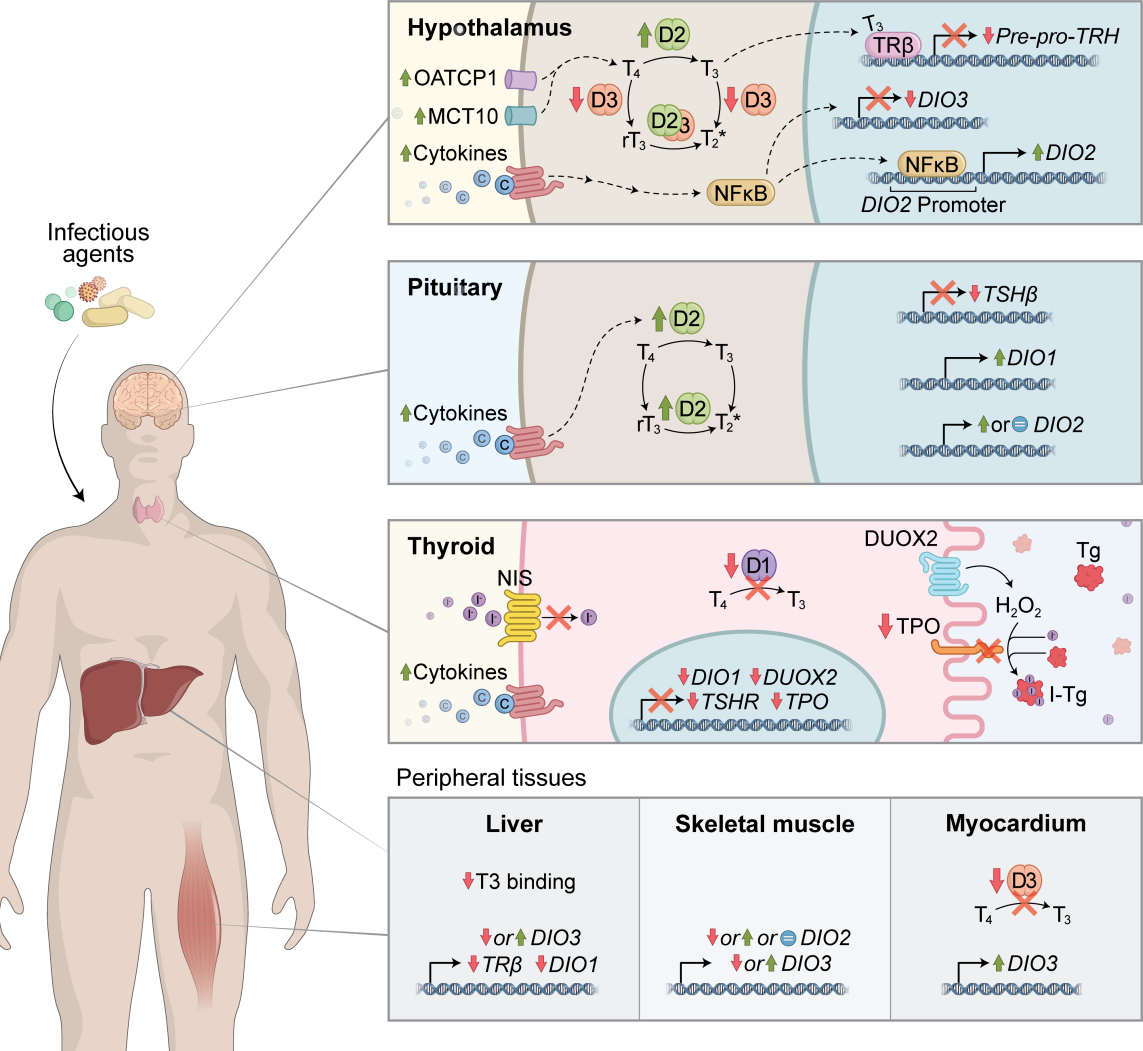
Mechanistic insights into COVID-19-induced Non-thyroidal illness syndrome (NTIS). Multiple mechanisms might be involved in the pathogenesis of NTIS. During severe illness, the hypothalamus-pituitary-thyroid (HPT) axis is profoundly affected, which is primarily mediated by pro-inflammatory cytokines. In the hypothalamus, illness promotes TRH downregulation due to abnormal T3-induced negative feedback. Potential mechanisms involved include increased local T3 production by D2 and increased expression in TH transporters (MCT10 and OATP1C1). At the pituitary level, illness impairs TSH response to low levels of circulating T_3_ and T_4,_ which can be secondary to reduced TRH levels or the direct effect of pro-inflammatory cytokines. Modulation of deiodinase activity might be additionally involved. Cytokines also inhibit multiple steps of TH biosynthesis and D1 activity in the thyroid gland, decreasing hormonal synthesis and secretion. In other peripheral tissues, suppressed D1 and increased D3 expression/activity could contribute to low T_3_ and high rT_3_ levels observed in sick individuals.

The entire hypothalamus-pituitary-thyroid (HPT) axis is profoundly affected by severe illness (**Figure 2**). At the hypothalamic level, TRH decrease is largely mediated by a T_3_-induced feedback mechanism in illness. The TH transporters MCT10 and OATP1C1 are upregulated in the hypothalamus of prolonged ill rabbits, which may further contribute to the negative feedback (64). Several subsequent animal studies using different illnesses models revealed a consistent upregulation of iodothyronine deiodinase 2 (D2) both in the PVN and in tanycytes, suggesting an increase in local T_3_ production, which helped explain the abnormal negative feedback resulting in TRH downregulation besides low levels of serum T_3_ (65, 66). Deiodinase 3 (D3) is negatively regulated by LPS in the PVN of mice and in cultured neuroblastoma cells, leading to increased intracellular T_3_ levels in these cells (67, 68). Although logical, the increase in intra-hypothalamic levels of T_3_ still lacks direct experimental evidence *in vivo*. At the pituitary level, illness leads to an impaired TSH response to low levels of circulating T_3_ and T_4_ (64) and lack of pulsatile secretion (69). This disturbed TSH response can be partially attributed to diminished TRH stimulus and to a direct effect of cytokines like IL-1β and TNF-α (70, 71). The contribution of pituitary’s D2 and/or D1 activity to these responses is still disputable.

The thyroid gland is also directly influenced by illness (**Figure 2**). Data from a vast collection of *in vivo* and *in vitro* models report that cytokines (e.g., IL-1α, IL-1β, TNF-α, IFNγ) can inhibit several steps of the TH synthesis machinery, including TSH receptor expression (72), iodide incorporation by NIS (73–75), iodide organification by TPO (76–78), DUOX expression (77), thyroglobulin production (79) and deiodinase 1 expression and activity (80–82). Collectively, these effects lead to a decrease in the synthesis and secretion of TH by the thyroid gland.Yet, most of the circulating T_3_ is not produced by the gland itself but through deiodination of T_4_ in peripheral tissues, such as liver, kidney and muscle (83, 84). During illness, alterations in the expression and function of D1 (85, 86), D2 (85, 87–89) and D3 (85, 86) might also contribute to serum TH abnormal levels (**Figure 2**).

There is still limited data on the literature outlining NTIS during viral infectious diseases. Although the few reports involve different populations and different viruses, they congregate in terms of clinical manifestations. In a cohort of HIV^+^ and AIDS patients, fT_3_ levels were lower than in healthy controls while calculated TBG capacity was increased (59). A subsequent study from Nigeria showed that, among 108 HIV-1^+^ individuals, 52% had abnormalities in thyroid function. Of these, 8.5% had subclinical hypothyroidism and 45.5% had NTIS. The HIV−1^+^ individuals had significantly lower TSH, T_3_ and T_4_ when compared with HIV-1^-^ controls (90).

Viruses that attack the respiratory tract have been also linked to the development of NTIS. The influenza A virus subtype H7N9 (A/H7N9) is a bird flu strain of the Influenza virus A (avian influenza) that infected humans in China in 2013. Of patients infected with H7N9, 70.6% presented abnormally low total T_3_ levels, 58.8% had low free T_3_ and TSH levels and 29.4% had abnormally low total and free T_4_ levels (below the lower limit of the reference ranges for each hormone) (91).

At the time of this publication, thyroid function tests of more than 2,000 COVID-19 patients have been reported in the literature. Collectively, they make clear that low serum fT_3_ and NTIS at admission strongly predict poor outcomes in these patients, although the utility of measuring fT_3_ at admission is still disputable in terms of cost-effectiveness since other biochemical indicators provide similar predictive value. Here we present some of the relevant clinical data regarding NTIS in COVID-19 patients.

Only three months after the beginning of the outbreak the first report of altered thyroid hormones in COVID-19 patients was presented in a retrospective analysis of 274 COVID-19 cases in the region of Wuhan. The authors revealed that serum levels of fT_3_ and TSH were significantly diminished in deceased patients compared to recovered patients, even though the decrease in TSH was still within the normal range (92). Similar observations were consistently reported in several other papers, although the incidence of decreased levels of fT_3_ and NTIS varied greatly, probably as a result of the huge discrepancies in the disease severity of the cohort included in each study.

In a study with 50 patients confirmed with moderate to critical COVID-19 with no history of thyroid disease, Chen et al. reported altered thyroid function in more than 60% of patients (93). Low TSH with or without lower-than-normal levels of tT_3_ were the most frequent alterations found in these patients. Interestingly, TSH and tT_3_ levels were lower in patients with COVID-19 when compared with healthy patients and non-COVID-19 pneumonia patients, suggesting that these clinical observations could be characteristic of SARS-CoV-2 infection. However, it is important to highlight that in this study thyroid function was evaluated while most of COVID-19 patients were under glucocorticoid treatment, which may affect thyroid function. In another cohort, Zhang and collaborators identified thyroid disorders in 28% of 71 COVID-19 patients, which included mainly NTIS (48%) and subclinical hypothyroidism (28%) (50). In agreement, NTIS was also found in more than 25% of COVID-19 in another Chinese cohort and its occurrence was associated with inflammation and disease severity (94).

A retrospective study published in October of 2020 conducted in Changsha (China) analyzed clinical and laboratory data of 149 patients with mild COVID-19 infection within the first 3 months of the pandemic and detected that 28% had NTIS, characterized by fT_3_<2.3 pg/mL and low or normal TSH. Compared to non-NTIS patients, NTIS-patients had lower tT_3_ (0.66 *vs*. 0.96 ng/mL, p<0.0001), tT_4_ (8.3 *vs*. 9.5 µg/dL, p<0.0001), and non-statistically significant lower TSH (1.36 *vs*. 1.74 µIU/mL, p = 0.06). Also, NTIS patients had a higher ESR, CRP and lymphopenia. NTIS was identified as an independent risk factor for disease severity by Cox-regression model (HR = 2.5 [95%CI 1.05-6.02]) and receiver operating characteristic (ROC) analysis (AUC = 0.81) (94). Another study searched for predictors of mortality in 121 ICU-admitted severe COVID-19 patients and detected that fT_3_ was the second-best predictor of death (AUC from ROC = 0.86), only after Sequential Organ Failure Assessment (SOFA) (AUC from ROC = 0.96) (95). Similar results were obtained in a cohort of moderately severe COVID-19 patients in which only 31.5% were admitted to the ICU (AUC from ROC = 0.84) (96).

Likewise, Gao et al. followed 100 COVID-19 patients (66% of then were severely or critically ill) and showed that fT_3_, TSH and fT_3_/fT_4_ ratio decreased with clinical deterioration and were lower in non-survivors. Moreover, the reduction in fT_3_ levels was independently associated with all-cause mortality (97). The prospective analysis of 115 COVID-19 patients in Italy, in which 18% had NTIS also observed that low fT_3_ was associated with mortality and inflammation. The following of these patients thought the hospitalization revealed that, not unexpectedly, the number of patients with decreased levels of fT_3_ and TSH increased during hospitalization. However, most of them were under corticosteroids therapy, emphasizing the need for cautious interpretation of TSH and TH after ICU admission (98).

A recent prospective analysis of 245 hospitalized patients with moderate or critical (ICU-admitted) COVID-19 patients in Brazil corroborated previous findings that fT_3_ is negatively associated with survival. Similar to previous findings, only 6.5% of patients had NTIS (characterized as fT_3_<2.0 pg/mL and low or normal TSH) but this diagnosis significantly increased the risk of death (OR = 7.05). This was the first study to measure rT_3_ levels in COVID-19 patients and it revealed that they were elevated (>0.35 ng/mL) in 63% of the patients and were significantly higher in critical patients compared to non-critical patients. Interestingly, although rT_3_ is frequently considered a hallmark of NTIS and NTIS increases the risk of death by COVID-19, serum rT_3_ levels were not associated to death risk, but the opposite. Patients with simultaneously fT_3_<2.6 pg/mL and rT_3_<0.38 ng/mL had a significantly worse clinical outcome (36% mortality rate) than patients with only fT_3_<2.6 pg/mL (17%), only rT_3_<0.38 ng/mL (20%), or none of these alterations (5%). Moreover, the authors showed that the value obtained from the mathematical product of T_3_ × rT_3_ had the strongest predictive value for mortality amongst all analyzed parameters (AUC from ROC = 0.7). Remarkably, the OR for death in patients with T_3_×rT_3_<1.29 was 8.08 (60).

These results shed light on a possible underappreciated protective role of rT_3_ in critically ill patients. Rastogi et al. (2018) evaluated the efficacy of intravenous rT3 administration as a neuroprotective agent in rat model of middle cerebral artery occlusion induced cerebral ischemia-reperfusion and in an *in vitro* model of oxygen glucose deprivation/reoxygenation. The authors demonstrated that the administration of rT3 significantly reduced markers of neuronal injury, oxidative stress [levels of malondialdehyde, glutathione and reactive oxygen species (ROS)], infarct size and neurological deficit after ischemic insult (99). The authors’ explanation would be the inhibition of rT3-induced DIO2 synthesis, with a decrease in T3 action at the brain level, reducing O2 consumption and oxidative stress. Interestingly, this finding contrasts with the prognostic value of rT_3_ in other conditions such as acute myocardial infarction, in which rT_3_ > 0.27 ng/mL increases the risk of 1-year mortality (HR = 3.0) (100). Of note, a rapid decrease in rT_3_ has also been observed in animal models of acute illness by turpentine-induced sterile abscess (67) and in critically ill HIV^+^ patients with or without secondary infections (59).

Consistent data has been obtained about the prognostic value of thyroid hormones abnormalities during COVID-19 illness and their impact on patient outcome. However, no advances have been obtained yet regarding the mechanisms accountable for fT_3_ reduction. More specifically, the contribution of thyroid hormone production and peripheral tissue TH metabolism (both through deiodination and minor metabolization routes) and also the role of TH metabolites like rT_3_. Increased rT_3_ is often observed in critically ill patients and is mostly attributed to decreased liver D1 activity and, with less certainty, to increased liver D3 (85). However, the finding by Beltrão et al. (2021) that decreased levels of rT_3_ leads to less favorable outcomes in COVID-19 raises the question of whether and how disturbances in TH metabolism in peripheral tissues may impact the course of the disease (60).

Recently, Beltrão et al. (2022) showed a protective role of the polymorphic variant DIO2 (Thr92Ala) heterozygous state in COVID-19 mortality. In his study, heterozygous in-hospital patients were protected by 47-62% of risk mortality. The protective role of Thr92Ala’s heterozygous advantage was supported in a meta-analysis of 21 studies on thousands of cases with different diseases, such as ischemic stroke, myocardial infarction, and left ventricular hypertrophy. This protection could be explained by the gene Thr92Ala-DIO2 expression association with endoplasmic reticulum stress, inflammation, oxidative stress, apoptosis, and mitochondrial dysfunction that are mechanisms also related to the pathophysiology of COVID-19 (101).

It must also be highlighted that even mild decreases in fT_3_ that remain within the normal range can indicate a poor clinical outcome. Schwarz et al. (2021) divided a small cohort of COVID-19 patients into fT_3_ tertiles and observed that the bottom tertile (that included patients below the normal range and in the lower part of the normal range) had a mortality rate 6-times higher than the two greater tertiles (96). Other studies also proposed fT_3_ cut-off values above the bottom limit of the normal reference range that efficiently indicated increased risk of death in COVID-19 (60, 95).

Several studies were published about COVID-19 patients and poor outcomes related to thyroid dysfunction during hospitalization. However, it is still unclear if the relation is accurate. To answer this question, we did a systematic review. We only selected studies with a sample of adults and greater than 50 subjects, excluding studies with children and pregnant women. After applying the eligibility criteria, we found 27 studies on the subject, counting 4554 patients. Our research focused on two main types of studies: (i) retrospective studies (**Table 1**) and prospective studies (**Table 2**). Most studies were retrospective (18 studies). The number of patients evaluated in the studies ranged from 50 to 506. Most patients were critical and had several comorbidities (mainly hypertension and DM), and the mortality rate ranged from 0 to 29%. In thyroid function assessment in-hospital, most studies evaluated the levels of TSH, fT4, and fT3 in their patients, while only two studies evaluated rT3 and thyroglobulin. Some patients were diagnosed with NTIS and thyrotoxicosis during hospitalization, and the prevalence ranged from 1.7-66.3% and 0-28%, respectively (**Tables 1**, **2**).

**Table 1:** Retrospective studies that analyzed thyroid function in COVID-19 patients during admission.

Author	Number of patients	Comorbidity	Severity	Thyroid function markers	Hyperthyroidism (%)	Main statistical findings	Thyroid function and clinical outcomes
Local Month/year	Men’s n (%) – Age	Length of stay	Mortality	Inflammatory markers	NTIS (%)	Limitations	
Wang et al	84	Not mentioned	Moderate 25% Critical 75%	TSH, TT3, TT4	Overt thyrotoxicosis 4% Subclinical thyrotoxicosis 4%	TT3 and TSH levels were significantly lower in COVID-19 patients (p < 0.001). Thyroid dysfunction was more commonly found in critical than in mild/moderate cases (74.6 vs 23.8%, p < 0.001). The group with thyroid dysfunction also had an increased level of leukocytes (p < 0.001), neutrophils (p < 0.001), CRP (p = 0.002), and PCT (p = 0.054); and a decreased level of lymphocytes (p < 0.001).	Thyroid dysfunction tended to be associated with longer viral nucleic acid cleaning time (14.13 ± 9.39 vs. 10.56 ± 8.29 days, p = 0.088).
China Feb/2021 (102)	53 (63.1%) 57.3 ± 14.5	Not mentioned	0%	Procalcitonin, PCR, IL-6, IL-10, TNF-α, interferon-γ	Not mentioned	Small sample size. Free T3, free T4, and reverse T3 were not measured.	
Gao et al	100	Not mentioned	Moderate 34% Critical 66%	TSH, FT4, FT3	Overt thyrotoxicosis 17%	FT3 levels are lower in severe ill patients (4.40 ± 0.88 vs 3.41 ± 0.90, p < 0.001). TSH levels are lower in severe ill patients (2.03 (1.24, 3.31) vs 1.20 (0.45, 2.05), p = 0.002).	The lower (versus upper) two-thirds of FT3 were associated with all-cause mortality HR (95% CI) of 9.23 (2.01, 42.28).
China Nov/2020 (97)	52 (52%) 66.1 ± 16	14 days ± 6	22%	PCR, D-dimer, IL-6, TNF-alfa, NT-proBNP	28%	Small sample size. Sample composed mainly of patients with severe COVID-19.	
Sun et al	336	Hypertension 35.3% Diabetes 15.3% CVD 9.3%	Mild/Moderate 92.3% Critical 7.7%	FT3, FT4, TT3, TT4	Not mentioned	TT3, FT3, TT4 and FT4 were significantly lower in moderate/critical patients; TT3 AUROC 0.96.	Thirty-six of the clinical and laboratory features analyzed were found to be statistically associated with severe/critical symptoms of COVID-19.
China Jul/2020 (103)	117 (34.8%) 50	Not mentioned	0%	CD3, CD4, CD19, CRP	Not mentioned	TSH and reverse T3 were not measured.	
Lania et al	287	Hypertension 49.5% Diabetes 24.4% CVD 14.3% COPD 12.2%	Critical (100% ICU)	TSH, FT3, FT4	Overt thyrotoxicosis 10.8% Subclinical thyrotoxicosis 19.9%	In the multivariate analysis, thyrotoxicosis was associated with higher IL-6 levels (odds ratio: 3.25, 95% CI: 1.97-5.36; P < 0.001). 16% of patients with overt thyrotoxicosis developed thromboembolic events.	The in-hospital mortality rate was higher in patients with either thyrotoxicosis or hypothyroidism. In discharged patients, the duration of hospitalization resulted to be significantly longer in cases with thyrotoxicosis as compared to those with either normal TSH or hypothyroidism.
Italy Oct/2020 (104)	193 (67.2%) 66 (27-92)	Not mentioned	21.4%	IL-6	Not mentioned	In several patients, thyroid function was assessed in the course of treatment with low-molecular-weight heparin.	
Sen et al	60	Not mentioned	Mild 43.3% Moderate 26.7% Critical 30%	TSH, FT3, FT4, TT3, TT4, TPOAb	Not mentioned	35% of the patients showed one or more abnormality in thyroid function. The commonest abnormality was low TSH, found in 11 patients (18.33%).	FT4 is associated with the severity of the disease (*P* = 0.009).
India Jan/2021 (105)	Not mentioned	Not mentioned	0%	Ferritin, D-dimer	Not mentioned	Small sample size.	
Chen et al	50	Not mentioned	Mild 30% Moderate 46% Critical 24%	TSH, FT3, FT4	56%	64% of the patients had abnormal thyroid function parameters.	The more severe the COVID-19, the lower the levels of TSH and TT3 (p <0.001).
China Jan/2021 (106)	33 (66%) 48,4 ± 13,7	Not mentioned	0%	Albumin	Not mentioned	Small sample size.	
Guo et al	121	Not mentioned	Critical 100%	FT4, FT3	Not mentioned	In the ROC curve, the FT3 variable was the best laboratory variable in predicting hospital mortality (AUC 0.863), 3.25 pmol/L cut-off.	Not mentioned.
China Jan/2021 (95)	57.0% 66 (56–72)	Not mentioned	28.9%	IL-2R, IL-6, IL-8, IL-10, TNF-α, NT-proBNP, troponin, and hs-CRP	Not mentioned	Small sample size. TSH and reverse T3 were not measured.	
Schwarz et al	54	Hypertension 38.8% Diabetes 33.3% CVD 29.6%	Moderated 68.5% Critical 31.4%	TSH, FT4, FT3	Not mentioned	Patients in the lowest FT3 tertile had significantly lower mean room air oxygen saturation on presentation (81%, 92.7%, and 93.7%, respectively; p = 0.006).	Patients in the lowest FT3 tertile had a significantly higher mortality rate (40%, 5.9%, and 5.9% in the first, second, and third tertiles, respectively; P = 0.008), more mechanical ventilation (45%, 29.4%, and 0.0%, respectively; P = 0.007), and ICU hospitalization (55%, 29.4%, and 5.9%, respectively; P = 0.006).
Israel Feb/2021 (96)	37 (68.5%) 58.7 ± 17.5	Not mentioned	10%	CRP, D-dimer, ferritin, troponin, LDH	Not mentioned	Small sample size. Reverse T3 was not dosed.	
Vassiliadi et al.	87	Not mentioned	Moderate 47.1% Critical 52.9%	TSH, FT4, TT3, TG	Overt thyrotoxicosis 6.9% Subclinical thyrotoxicosis 6.8%	T3 and TSH levels were lower in the ICU patients (70.5 ± 31.9 vs 89.7 ± 42.0, *P* = 0.001 and 0.95 ± 0.93 vs 1.66 ± 1.46, *P* ≤ 0.001, respectively).	The prevalence of thyroid hormone abnormalities increased with increasing disease severity.
Greece Jun/2021 (107)	69 (66.3%) 59.3 ± 18.3	Not mentioned	14.9%	IL-6	47.1%	Small sample size. Free T3 and reverse T3 were not measured.	
Yazan et al	205	Hypertension 42.6% Diabetes 26.3% CVD 15.2% COPD 12.3% Neoplasia 5.8%	Moderate 85% Critical 15%	TSH, FT3, FT4, TGAb, TPOAb	Overt thyrotoxicosis 3.9% Subclinical thyrotoxicosis 4.3%	Thyroid dysfunction rate was 65.8% in this study. FT3 (rho = -0.34, *p* < 0.001), and TSH (rho = -0.21, *p* = 0.002) had weak negative correlations with WHO illness severity scores.	Length of hospitalization, rate of oxygen demand, ICU admission and mortality were lower in euthyroid patients. FT3 and TSH levels were significantly lower in patients admitted to ICU (p < 0.001 and p = 0.005, respectively).
Turkey Aug/2021 (108)	113 (55.1%)	Not mentioned	4.3%	CRP, D-dimer, ferritin, DHL	52.6%	Absence of a control group.	
Ahn J et al.	119	Hypertension 52.1% Diabetes 30.3% CVD 18.4% COPD 5.9%	Moderate 26.9% Critical 73.1%	TSH, FT3, FT4	Overt thyrotoxicosis 0% Subclinical thyrotoxicosis 14.3%	Patients with severe to critical COVID-19 disease had lower TSH (median: 0.90 mIU/L vs 1.67 mIU/L, *p* = 0.006) and T3 (median: 0.82 ng/mL vs 1.11 ng/mL, *p* < 0.001) levels compared with those with non-severe disease. T3 was negatively correlated with hs-CRP (*r*=−0.373, *p* < 0.001) and WBC count (*r*=−0.463, *p* < 0.001).	COVID-19 patients in the lower third of T3 levels (compare to middle and upper third of T3 levels) had poor outcomes: ICU admission (61.5% vs 32.5% vs. 30%, p = 0.005), mechanical ventilation (46.2% vs 27.5% vs 12.5%, p = 0.001), and death (48.7% vs 32.5% vs 5%, p < 0.001). The Kaplan-Meier curves for survival showed increased mortality of the lowest third T3 (log-rank P=0.014).
Korea Aug/2021 (109)	62 (52.1) 64.3 ± 16.8	Not mentioned	28.6%	CRP	18.5%	Sample composed mainly of patients with severe COVID-19. Reverse T3 was not measured.	
Clausen et al.	116	Hypertension 46% Diabetes 33% Asthma 10% COPD 8%	Moderate 83% Critical 17%	TSH, FT4	Overt thyrotoxicosis 1.7% Subclinical thyrotoxicosis 9.5%	18.1% patients had biochemically thyroid dysfunction. IL-8 (r = –0.248, P = 0.008), IL-10 (r = –0.253, P = 0.007), IL-15 (r = –0.213, P =0.02), IP-10 (r = –0.334, P = 0.0003) and GM-CSF (r =–0.254, P =0.007) were inversely correlated with TSH. IL-8 levels, IP-10, and GM-CSF were higher in patients with serum TSH < 0.4 mIU/L.	Neither TSH in the whole cohort nor in the group with TSH levels <0.4 mIU/L was associated with 30- and 90-day mortality in crude and adjusted logistic regression models (adjusted for age, sex, and IL-6).
Denmark Set/2021 (110)	44 (38%)	Not mentioned	24%	35 cytokines	1.7%	Small sample size. Free T3 and reverse T3 were not measured.	
Okwor et al	90 (45 control)	Not mentioned	Not mentioned	TSH, FT3, FT4	Overt thyrotoxicosis 2.2%	Plasma levels of FT3 (4.19 ± 1.32 vs 2.42 ± 0.83) and TSH (2.60 ± 1.04 vs 1.68 ± 0.67) were significantly higher in COVID-19 patients compared to healthy controls (p< 0.001).	Amongst COVID-19 patients 7 (15.6%) presented euthyroid sick syndrome whereas no cases were found in the control group.
Nigeria Set/2021 (111)	34 (75.6%) 35.3 ± 12.4	Not mentioned	0%	CPR	15.6%	Small sample size. Young population and mostly men. Severity criteria were not used.	
Dutta et al	236	Hypertension 43.2% Diabetes 50.4% Hypothyroidism 18% CVD 8%	Moderate 94.1% Critical 5.9%	TSH, FT3, FT4	Subclinical thyrotoxicosis 3,8%	Low FT3, high TSH and low TSH were seen in 56 (23.7%), 15 (6.4%) and 9 (3.8%) patients, respectively.	Cox regression analysis showed that low FT3 was associated with severe COVID-19 (P =0.032, HR 0.302; CI 0.101–0.904). The duration of hospital stay correlated negatively with both FT3 and TSH.
India Nov/2021 (112)	159 (6%) 54 (15-91)	Eight days (1-44)	4.7%	CPR, D-dimer, IL-6, ferritin, DHL.	23,7%	Most patients with moderate disease.	
Lang et al	127	Hypertension 41.7% Diabetes 21,3% CVD 10.2% COPD 10.2%	Mild 44.1% Moderate 42.5% Critical 13.4%	TSH, FT4, FT3	Not mentioned	The serum levels of TSH [0.8 (0.5–1.7) *vs*. 1.9 (1.0–3.1) μIU/mL, *P* = .031] and FT3 [2.9 (2.8–3.1) *vs*. 4.2 (3.5–4.7) pmol/L, *P* <.001] were lower in non-survivors than in survivors.	Patients with low FT3 (<3.1 pmol) had a higher risk of death (adjusted OR 13.2, 95% CI 3.87–55, p < 0.001).
China Nov/2021 (113)	62 (48.8%) 66 (53-71)	Not mentioned	8.6%	CRP, D-dimer, IL-6.	16,5%	Small sample size. Corticosteroids in the treatment of COVID-19.	
Zheng et al.	235	Hypertension 35.3% Diabetes 15.3% DCV 9.3% COPD 5.9%	Moderate 20.8% Critical 79.2%	TSH, FT3, FT4	Not mentioned	The proportion of subclinical hypothyroidism was 7.23% in COVID-19 patients. Patients with NTIS had higher CRP (17.6 (2.6) vs 67.4 (7.4), p<0.001), WBC count (6.26 (0.2) vs 7.59 (0.6), p=0.001) and ESR (43.9 (2.7) vs 81.5 (8.5), p<0.001).	Patients with NTIS had higher incidences of COVID-related complications, including ARDS (9.1–13.0% vs 0.0–1.1%), acute cardiac injury (54.5–70.0% vs 15.3–23.5%), acute kidney injury (21.7–27.3% vs 0.0–2.7%), shock (36.4 47.8% vs 0.0–1.6%), hypoalbuminemia (45.5–52.2% vs 18.6– 23.5%), and coagulopathy (27.3–30.0% vs 0.0–10.9%), as well as higher severe types of COVID-19 (100% vs 75.5–76.5%) compared to patients with normal thyroid function. .
China Nov/2021 (114)	112 (47.6%) 61 (51-69)	Not mentioned	6.8%	PCR, D-dimer, IL-6, BNP	14.47%	Most critically ill patients. Reverse T3 was not measured.	
Sethi et al	57	Not mentioned	Mild 33.3% Moderate 33.3% Critical 33.3%	TSH, FT3, FT4	Overt thyrotoxicosis 28% Subclinical thyrotoxicosis 9%	28% of the patients had raised T4 and around 9% had decreased TSH. A negative correlation was found between TSH and CRP (r=-0.541).	T3 (H = 11.98, p =0.02) and T4 (H = 6.71, p = 0.035) were lower in higher disease severity (p <0.05).
India May/2022 (115)	39/57 (68%) 47.1	Not mentioned	Not mentioned	CPR	Not mentioned	Small sample size.	
Okoye et al	95	Not mentioned	Mild 55.4% Moderate 19.3% Critical 25.3%	TSH, FT3, FT4	Not mentioned	There is no difference in the incidence of NTIS between patients with COVID-19 (66.3%) and patients with non-COVID-19 pneumonia (67,9%) (*p* = 0.82).	Among COVID-19 patients, a slightly lower mortality of NTIS patients was observed (23.8% vs 31.2% respectively, *p*=0.43), while non-COVID-19 patients with NTIS showed a three times higher mortality than non-NTIS (14.5% vs 3.8% respectively, *p*=0.09).
Italy May/2022 (116)	52.6% 81.9 ± 7.8	Not mentioned	26.3%	CPR	66.3%	Small sample size. Only hospitalized elderly.	

**Table 2:** Prospective studies that analyzed thyroid function in COVID-19 patients during admission.

Author,	Number of patients	Comorbidity	Severity	Thyroid function markers	Hyperthyroidism (%)	Main statistical findings
Local Month/year	Men’s n (%) – Age	Length of stay	Mortality	Inflammatory markers	NTIS (%)	Limitations
Muller et al.	145 (COVID-19), 93 (ICU), 52 (non-ICU); 101 (non-COVID-19)	Not mentioned	Moderate 35.9% Critical 64.1%	TSH, FT4, FT3	Overt thyrotoxicosis 11.8% Subclinical thyrotoxicosis 17.7%	FT4 were higher in the COVID-19 ICU group (18.7 ± 5.4) than in the COVID-19 NICU (13.5 ± 4.6) group (p=0·016) but not in non-COVID-19 group (16.2 ± 2.4) (p=0·38).
Italy Set/2020 (117)	89 (61.4%) COVID-19 ICU 65.3 ± 12.9 years COVID-19 non-ICU 70.3 ± 18.1 years	COVID-19 ICU (23.8 ± 15.8 days) COVID-19 NICU (22.3 ± 15.5 days)	COVID-19 ICU (18.7%) COVID-19 NICU (7.8%)	PCR, D-dimer, ferritin, DHL.	Not mentioned	Small sample size. Reverse T3 and other biomarkers were not measured.
Khoo et al.	334	Hypertension 48,5% Diabetes 39,5% CVD 23,7% COPD 17,4% CKD 13,2%	Moderate 89,3% Critical 10,7%	TSH, FT4	Overt thyrotoxicosis 0% Subclinical thyrotoxicosis 5.4%	Patients with COVID-19 had lower TSH (1.03 mU/L) and FT4 (12.60 pmol/L) than patients without COVID-19: TSH (1.48 mU/L, P = 0.01) and T4L (13.11 pmol/L, P = 0.01).
United Kingdom Jan/2021 (118)	203 (60,8%) 66,1 ± 16 years	8 days (IQR 6-11).	26%	CPR, cortisol, albumin	Not mentioned	Free T3 was not measured; therefore, patients with NTIS were not analyzed.
Guven et al.	250	Not mentioned	Moderate 50% Critical 50%	TSH, FT4	Overt thyrotoxicosis 4% Subclinical thyrotoxicosis 5.2%	The FT3 level showed a negative correlation with length of hospital stay and CRP (r = −0.216, p=0.001; r = −0.383, P < 0.0001).
Turquia Mar/2021 (119)	157 (63%) 68 (54-78) years	9 days (IQR 5-15).	15,2%	PCR, D-dimer, ferritin	13%	Small sample size. Diabetic and nephropathy patients were excluded.
Lui et al	367	Hypertension 24,3% Diabetes 16,3% CVD 5,4% CVA 2,7% COPD 3,5%	Mild 75,2% Moderate 21% Critical 3,8%	TSH, FT3, FT4	Subclinical thyrotoxicosis 8,2%	Patients with NTIS had a higher risk of death (adjusted OR 3.18, 95% CI 1.23–8.25, p = 0.017),
China Apr/2021 (120)	172 (46,9%) 54 ± 15 years	8 days (IQR 6-13).	1%	CPR, CPK, TGP, DHL	7,4%	Most mild COVID-19 patients. Reverse T3 not measured.
Campi et al	115	Hypertension 64% Diabetes 17,5% Cardiopathy 6,3% CVA 4,2% Pneumopathy 3,1%	Critical 100% (ICU)	TSH, FT3, FT4, Tg, anti-Tg	Subclinical thyrotoxicosis: during admission (10,4%), during hospitalization (23,5%)	Low TSH levels were found either at admission or during hospitalization in 39% of patients, associated with low FT3 in half of the cases. In the univariate analysis, the predictors of mortality were low FT3 (P <0.0001) and low FT4 (P = 0.01).
Italy Mai/2021 (98)	97 (67%) 68.1 ± 14 years	21 ± 19 days	31.3%	PCR, Cortisol	9%	Only severe COVID-19 patients.
Beltrao et al	245	Hypertension 66.5% Diabetes 44.6% DCV 13.8% Pneumopathy 4.4%	Non-critical 73.9% Critical 26.1%	TSH, FT3, FT4, TT3, rT3, Tg anti-Tg	Subclinical thyrotoxicosis 27.3%	fT3 levels were lower in critically ill compared with non-critical patients [fT3: 2.82 (2.46–3.29) pg/mL vs. 3.09 (2.67–3.63) pg/mL, p = 0.007]. Serum reverse triiodothyronine (rT3) was mostly elevated but less so in critically ill compared with non-critical patients [rT3: 0.36 (0.28–0.56) ng/mL vs. 0.51 (0.31–0.67) ng/mL, p = 0.001]. There is correlation between in-hospital mortality and serum fT3 levels (odds ratio [OR]: 0.47; 95% confidence interval [CI 0.29–0.74]; p = 0.0019), rT3 levels (OR: 0.09; [CI 0.01–0.49]; p = 0.006) and the product fT3 · rT3 (OR: 0.47; [CI 0.28–0.74]; p = 0.0026).
Brazil Nov/2021 (60)	145 (59.1%) 62 (49-75) years	6 (4–10) days	16.7%	PCR, D-dimer, fIL-6, DHL, albumin	6.5%	It is unclear whether a decrease in caloric intake, a weight loss, or a combination of these factors are the cause of decreased fT3 levels in COVID-19 critically ill patients.
Vizoso et al	78	Hypertension 55.1% Diabetes 25.6% CVD 15.4% COPD 12.8% Cancer 11.5%	Critical 100%	TSH, FT3, FT4, T3, rT3	Not mentioned	FT3 levels were lower in non-survivors (1.6 ± 0.2) vs survivors (1.8 ± 0.5) p = 0.02.
Spain Nov/2021 (121)	55/78 (70.5%) Survivors 59 ± 12 Non-survivors 68 ± 12	Survivors 37 (22–83) days Non-survivors 18 (7–39) days	29.5%	Not evaluated	46.2%	Small sample size. Critical patients only.
Ilera et al.	55	Not mentioned	Mild 22% Moderate 27.1% Critical 50.8%	TSH, FT3, TT3, FT4, TT4, anti-TPO	0%	The T3/T4 ratio was significantly lower in patients with severe disease compared with those with mild/moderate infection [7.5 (4.5–15.5) vs. 9.2 (5.8–18.1); p =0.04] and lower in patients who died than in patients who were discharged [5.0 (4.53–5.6) vs. 8.1 (4.7–18.1); p = 0.03]
Argentina Dez/2021 (122)	28 (50.9%) 56 (21-89) years	Not mentioned	7.4%	CPR, D-dimer, ferritin, DHL, VHS, fibrinogenin	54.5%	Small sample size.
Sparano et al	506	Hypertension 51.3% Diabetes 17% CVD 26.9% COPD 7.1% Cancer 18.4%	Mild/Moderate 73.7% Critical 26.3%	TSH, FT3, FT4	Overt thyrotoxicosis 12.4%	In Kaplan–Meier and Cox regression analyses, fT3 was independently associated with poor outcome and death (*p* = 0.005 and *p* = 0.037, respectively). A critical fT3 threshold for levels < 2.7 pmol/l (sensitivity 69%, specificity 61%) was associated with a 3.5-fold increased risk of negative outcome (95%CI 2.34–5.34).
Italy 2022 (123)	62.3% 68.8 ± 1.6 years	12.5 ± 9.1 days	19%	IL-6, NT-ProBNP, PCR, procalcitonin, D-dímer, DHL,	57%	Monocentric study, without a control group. Most mild patients. Reverse T3 levels were not evaluated.

The abnormalities in TH and TSH in COVID-19 seem to be, as for other critical illnesses, transient. Khoo et al. (124) compared the levels of T_4_ and TSH at admission and after COVID-19 recovery with the patient-matched baseline level assayed in 2019 (i.e., before the pandemic) and confirmed that after recovery serum hormone levels returned to baseline. The lack of knowledge about the precise role of thyroid hormone fluctuations during illness makes it challenging to estimate the potential of a TH replacement therapy in COVID-19. An ongoing randomized placebo-controlled clinical trial (NCT04348513) aims to investigate whether the administration of T_3_ (liothyronine, 0.8g/kg i.v.) to ICU-admitted COVID-19 patients alleviates their need for cardiorespiratory support (54).

Importantly, most of the above-mentioned studies presented clinical data of patients admitted throughout the first 6 months of 2020, when SARS-CoV-2 variants of concern (VOC) were still not widespread. Whether the clinical course of patients infected by VOC will differ from the course of the disease caused by the original strain is still to be elucidated.

### 4.2 Subacute thyroiditis

**Figure 3:**
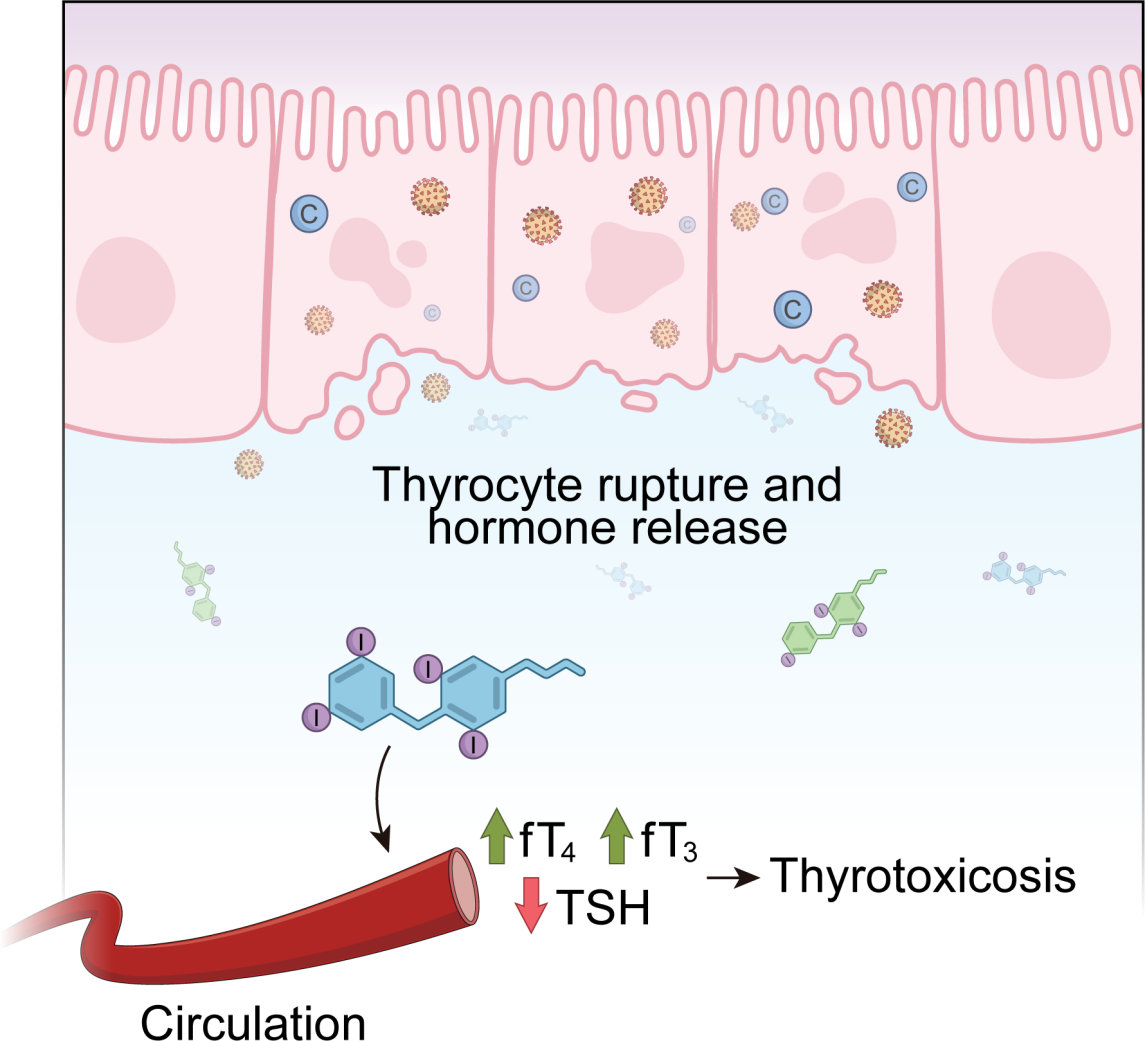
The course of Subacute Thyroiditis (SAT). Subacute Thyroiditis is a self-limited inflammatory disorder of the thyroid gland that often follows a viral infection. The damage and rupture of follicular cells, caused by the inflammatory process, results in the release of T_3_ and T_4_ in circulation, inducing thyrotoxicosis symptoms in the first weeks of disease. When extensive follicular destruction occurs, subacute thyroiditis can progress to hypothyroidism before returning to the euthyroid state.

Subacute Thyroiditis (SAT), also known as De Quervein’s thyroiditis or subacute granulomatous thyroiditis, is a self-limited inflammatory disorder of the thyroid gland that usually disappears in a few months. Patients usually show neck pain and enlarged thyroid and tenderness upon palpation. Symptoms as low fever, fatigue, malaise and myalgia are common (125). The development of subacute thyroiditis has been linked to viral infection or post inflammatory reaction to several different viruses, including mumps virus, measles virus, rubella virus, adenovirus, cytomegalovirus, enterovirus, Coxsackie virus, HIV, influenza virus and dengue fever virus (126). The inflammatory process causes damage and rupture of follicular cells, which leads to the release of T_3_ and T_4_ in circulation, inducing thyrotoxicosis symptoms in the first weeks of disease (**Figure 3**).

A study published in 1967 demonstrated that 45% of patients with subacute thyroiditis presented an increase of at least four times in viral antibodies during their thyroid disease (127). Curiously, clusters of subacute thyroiditis have been reported during outbreaks of viral infection (126) and a higher prevalence of this disease has been reported during the summer, which is the season of the highest incidence of enteroviruses and enterobacteria (128). It is well known that infections can cause direct effects as tissue damage, and also non-infectious consequences, such as malignancies, immunodeficiency syndrome, peptic ulcer and autoimmune diseases. It has been demonstrated that several infectious agents are involved in the development of autoimmune diseases, such as rheumatic fever, lupus erythematosus, insulin-dependent diabetes, among others (129). In this context, thyroid diseases thought to be of infectious etiology *(e.g.* subacute thyroiditis) have been shown to be associated with thyroid autoimmune phenomena. When follicular destruction is extensive, subacute thyroiditis can progress and cause hypothyroidism (Hashimoto’s thyroiditis) (**Figure 3**). In fact, thyroid autoantibodies (anti-thyroglobulin and anti-thyroid peroxidase) have been found in 40-60% of patients with subacute thyroiditis (127, 130). Around 30% of those patients will experience subsequent hypothyroidism before returning to euthyroid state (125). There are also reports of the sequential occurrence of Graves’ disease and subacute thyroiditis (131, 132).

Several mechanisms have been proposed for induction of thyroid autoimmunity by viral agents including: (1) viral induction of changes in self antigen expression, or exposure of cryptic epitopes; (2) induction of local inflammation (e.g. by cytokine release), resulting in activation of autoreactive T-cells (bystander mechanism); (3) molecular mimicry between viral antigens and thyroidal antigens; (4) induction of heat shock proteins in the thyroid; and (5) induction of aberrant expression of MHC class II molecules on thyroid cells (129). However, these autoimmune phenomena seem to represent a non-specific and transient response to the inflammatory release of thyroid antigens and classical autoimmune thyroid disease is only rarely triggered by viral infections (128).

Viruses that attack the respiratory tract have been linked to the development of thyroiditis. Cases of De Quervain thyroiditis, with low TSH levels and high levels of free T_3_ and T_4_, were described in the course of H1N1 influenza infection (133, 134). The H1N1 virus has also been linked to the occurrence of thyroid storm, which is a potentially fatal intensification of thyrotoxicosis, and is characterized by hyperthermia, tachycardia, severe agitation and altered mental status (135, 136). In 2009, a 31-year-old female diagnosed with community-acquired bronchopneumonia with possible influenza A (H1N1) viral pneumonia, presented tachycardia and T_3_ levels 16 times higher than the threshold. The diagnosis of thyroid storm was made, but despite treatment with propylthiouracil (PTU), the patient progressed from multiorgan failure to brain death (135). A recent case report also presented the occurrence of a thyroid storm associated with Influenza A infection in a 10-year old girl (137).

A prospective study followed 61 survivors of the 2002 SARS epidemy (with no pre-existing endocrine diseases) 3 months after recovery and observed that 6.7% of them became biochemically hypothyroid (with one case of primary and three of central hypothyroidism) and 39% had hypocortisolism (two of hypocortisolic patients had also transient subclinical thyrotoxicosis). The authors speculated that these effects might be due to SARS-induced reversible hypophysitis or a direct effect of the virus on the hypothalamus (138). In addition, in subjects who died of SARS, follicular epithelial damage in the thyroid gland were found during the autopsy, with large numbers of cells exfoliated into the follicle and undergoing apoptosis (139). A subsequent study also observed in autopsies that the adenohypophysis of SARS patients had profound alterations including lower positive-cell count and staining intensity for TSH, GH and ACTH (140).

In May of 2020, an Italian case-report provided the first case of subacute thyroiditis potentially associated with a prior mild COVID-19 infection (141). An 18-year-old female patient reported neck pain radiated to the jaw, fever and palpitation 15 days after a positive RT-PCR for SARS-CoV-2. The patient showed painful and enlarged thyroid to palpation and laboratory findings typical of acute phase of destructive thyroiditis, including elevated fT_3_ and fT_4_, undetectable TSH, detectable thyroglobulin (Tg) and anti-Tg antibodies. Antibodies against TPO and TSH receptor were absent, and the inflammatory markers CRP, ESR and white blood count were elevated. Neck ultrasound revealed diffuse hypoechoic areas. The patient was diagnosed with SAT and treated with prednisone. Forty days after diagnosis thyroid function and inflammatory markers were normalized. Subsequent studies also reported additional isolated cases of painful symptomatic SAT developed 16 to 42 days after COVID-19 infection (142, 143) and also cases during active COVID-19 disease (144–147), reinforcing a possible association between SARS-CoV-2 infection and SAT (**Table 1**).

The THYRCOV study retrospectively evaluated the thyroid function in a cohort of 287 patients hospitalized for COVID-19 in non-intensive care units and found that thyrotoxicosis was the thyroid disorder with higher prevalence (104). In this cohort, around 55 patients (20.2%) showed low TSH (≤0.10 mU/L), from whom 31 patients presented overt thyrotoxicosis. From those 31 patients, 10 had atrial fibrillation. Fifteen patients (5.2%) were diagnosed with high TSH (>4.80 mU/L), and overt hypothyroidism was present in two of those patients (104). Thyrotoxicosis was significantly associated with higher serum IL-6 levels. Thyroid function was spontaneously improved during the follow-up, which raised the hypothesis that the thyrotoxic state could be related to destructive thyroiditis, but it was not properly investigated in this cohort.

Corroborating this findings Muller and colleagues found a thyrotoxicosis’ (TSH <0.28 mU/L and/or fT_4_>1.7 ng/dL) prevalence of 15% (13 in 85 patients) in patients with COVID-19 (HICU-20) admitted in high intensity of care units (HICUs), compared to 2% (2 in 41 patients) in those admitted in low intensity of care units (LICU-20) and only 1% (1 of 78 patients) in patients admitted in HICUs in 2019 for non-COVID-19 related reasons (HICU-19). Free T_3_ equally low in all groups. TSH levels were lower in HICU-20 than other groups, while fT_4_ was higher in HICU-20 only when compared to LICU-20 (117). However, it is important to mention that fT_4_ and fT_3_ were only measured in patients whose TSH levels were less than 0.45 mIU/L. Since a greater proportion of HICU-20 group (24,7%) showed low TSH levels (TSH <0.45 mIU/L) when compared to HICU-19 (17,7%) and LICU-20 (9,8% groups), TH levels were more frequently measured in HICU-20, which might be a bias. CRP levels were higher in COVID-19 patients than the non-COVID-19 group. From the 6 patients with thyrotoxicosis that were followed post-discharge, all had normal thyroid function 1.5-2 months later and were negative to thyroid autoantibodies and 3 of them had ultrasound and CT scans suggestive of subacute thyroiditis. However, since no patient reported neck pain, TSH levels were not extremely low, fT_4_ was not very high and lymphopenia and not leukocytosis were present, it was probably not a typical case of subacute thyroiditis. The authors suggested a routine evaluation of thyroid function in patients in ICU due to increased risk of thyrotoxicosis.

A Chinese cohort of 367 patients with predominantly mild to moderate COVID-19 detected abnormal thyroid function in 62 patients (16,9%) (120). Twenty-seven patients (7,4%) had non-thyroidal illness syndrome (NTIS) and 30 patients (8,2%) had biochemical alterations that were suggestive of distinct phases of thyroiditis such as: isolated low TSH and high-normal fT4, isolated slightly elevated fT_3_, high-normal Ft4 or isolated low fT4. None had overt thyrotoxicosis. Of these 30 patients with subnormal TSH, 5 presented anti-TPO or anti-TSHR autoantibodies, suggesting an autoimmune component in these cases. Pre-existing autoimmune thyroid disorder was present in 5 patients.

In contrast with the previous studies, Khoo and collaborators, did not found any case of overt thyrotoxicosis in a cohort 334 patients admitted with COVID-19 in intensive therapy unit, either during the disease or follow-up (124). Most COVID-19 patients (86.6%) were euthyroid but 5.7% present subclinical hyperthyroidism and a small proportion present overt hypothyroidism (0,6%), which did not differ from non-COVID patients. A small significant reduction in TSH and fT_4_ was observed in patients with COVID-19 when compared with non-COVID-19 patients which might be compatible with a nonthyroidal illness syndrome and did not justify any treatment. A negative correlation between TSH and Cortisol or CRP was observed.

Of note, since the beginning of COVID-19 vaccination campaigns, some studies show the development of thyroid disease, especially thyroiditis, after the vaccines administration. Currently, more than 30 articles reporting data of SAT onset after COVID-19 vaccine. This relation seems more prevalent in women and the main symptoms are neck pain, palpitations, fatigue, fever and weight loss (148, 149). The patients almost always presented with thyrotoxicosis and elevated serum inflammatory markers (148, 150). Two cases of vaccine-induced Graves’ disease have been reported in two female health-care workers of 28 and 40 years of age. The patients presented typical symptoms of thyrotoxicosis 2-3 days after receiving the first dose of the Pfizer-BioNTech vaccine (151). A sistematic review concluded that the thyroid disease onset occurred an average of 11 days after the administration of the vaccine (150). Cases of SAT had already been observed after the administration of the H1N1 vaccine (152, 153). The first mechanism suggested to explain the relation between COVID-19 vaccination and thyroid disease is the autoimmune/inflammatory syndrome induced by adjuvants (ASIA) (148, 151). But it is also already shown that antibodies against SARS-CoV-2 proteins could cross-react with tissue antigens, including thyroid peroxidase (TPO) (154). Despite that, considering that billions of vaccines against COVID-19 have already been administered around the world, the development of thyroid disorders following SARS-CoV-2 vaccination is a very rare side effect.

### 4.3 Thyroid features in COVID-19 patients

In 2002, the Severe Acute Respiratory Syndrome (SARS) caused by SARS-CoV, a member of the Coronaviridae family, became an epidemic and rapidly spread to 26 countries (155). Extensive follicular damage, with large numbers of cells exfoliated in the follicle, was observed in the thyroid glands obtained from five SARS patients. The follicular architecture was prominently affected, showing follicular distortion and collapse (139).

In the first report of subacute thyroiditis (SAT) associated with COVID-19 infection, diffuse hypoechoic areas in the thyroid ultrasound were reported, in addition to the alterations in FT3, FT4, TSH, and the presence of TgAb (142). Subsequently, other studies found alterations in the thyroid ultrasonography of COVID-19 patients that developed SAT, including bilateral hypoechoic areas (145–147, 156), heterogeneity in the parenchyma (144), a relative diffuse decrease of vascularity (144, 146, 147) and increased vascularity (145) and inflammation (104). Thoracic computed tomography also showed that COVID-19 patients present altered thyroid tissue density during their infective states compared to prior infection. In these patients, the iodine content in thyroid tissue decreased, suggesting thyroiditis (157). Likewise, a case report of SARS-CoV-2-associated thyroid storm also detected ultrasound changes in the thyroid. The patient, a 25-year-old woman, presented exophthalmos, tachycardia, diffusely enlarged goiter with a bruit, and fine tremor. Laboratory results demonstrated very low TSH levels (TSH<0.01 mIU/L) and high levels of FT4 (5.34 ng/dL) and TT3 (654 ng/dL). Thyroid ultrasound revealed heterogeneous echotexture with increased vascularity (158).

As discussed before, viral infections are considered a major factor in the pathogenesis of autoimmune thyroid diseases, and a link between SARS-CoV-2 infection and Hashimoto thyroiditis and Graves’ disease has already been reported. In a patient who started to present hyperthyroidism symptoms (fatigue, shortness of breath, palpitations, and weight loss) nearly 3 weeks after a mild SARS-CoV-2 infection, the ultrasound showed a diffusely heterogeneous and irregular thyroid and a nodular image below the sternal notch. Thyroid scintigraphy confirmed Graves’ disease pattern (159). Not many studies have evaluated the *post-mortem* thyroid of patients who died of COVID-19. Of these, some found no significant damage of follicular thyroid cells (102), while others reported chronic inflammation of the thyroid, follicular epithelial cell disruption, or interstitial lymphocytic infiltration (160, 161).

Importantly, thyroid morphological changes persist even after COVID-19 resolution. A study conducted in China evaluated the thyroid of 79 COVID-19 survivors approximately one month after acute COVID-19 infection. At this time, all patients presented normal T4, T3 and TSH levels. Interestingly, higher SARS-CoV-2 viral load on presentation was associated with smaller thyroid volumes among the male survivors. The authors also observed that 13.9% of the COVID-19 survivors had ultrasonographic features suggestive of thyroiditis (5 had heterogeneous echogenicity, 6 had abnormal vascularity and 3 had micro-nodulation) (162). A similar result was also found in patients whose thyroid was evaluated 6 months after COVID-19 infection. In the Turkish cohort, the mean thyroid gland volume was significantly lower in COVID-19 survivors (10.3 ± 3.4 mL) than in non-COVID patients (14 ± 5.3 mL). There were no differences in thyroid gland volume between males and females (163). These findings encourage longitudinal follow-up to clarify a possible direct viral effect of thyroid atrophy.

## 5 COVID-19 and thyroid: State-of-art

Herein, we present an overview of the current knowledge regarding the relationship between thyroid dysfunction and SARS-CoV-2 infection. Overall, these data revealed that abnormal thyroid function may occur during and in the convalescence post-COVID condition phase. Although the cellular and molecular mechanisms are not completely understood, the evidence suggests that the “cytokine storm” is an important mediator in this context. It is very likely that indirect mechanisms (e.g., increased serum cytokines and immune cells) are responsible for most of the effects observed in the whole HPT axis. On the other hand, some authors have also proposed that the thyroid cells could be directly infected by SARS-CoV-2. It has been consistently demonstrated in multiple datasets that ACE2 mRNA is expressed in both human thyroid tissues and primary cultured cells, suggesting that the thyroid could be vulnerable to direct viral infection and its cytopathic effects (21, 164). However, stringent immunohistochemistry analysis from the The Human Protein Atlas performed with 7 different antibodies against human ACE2 reveals that thyrocytes do not have ACE2 protein. Indeed, ACE2 protein has been detected in endothelial cells within the thyroid gland, which may explain the detection of ACE2 mRNA in whole tissue extracts. Hence, thyroid function alteration during COVID-19 is more likely a result of pro-inflammatory signals and impaired central control than a direct infection of follicular cells by SARS-CoV-2.

However, it is important to highlight that the studies found in the literature have limitations. First of all, they were retrospective and, in most of them, thyroid function tests were performed only at admission and/or days after resolution, which did not allow the observation of dynamic alterations in thyroid function during disease progression. Some studies did not assess thyroid function on all cohort, while others measured only TSH or limited fT_3_ or fT_4_ measurements only to patients with abnormal TSH. Moreover, only one study measured rT_3_ levels. Thus, future studies are needed to better investigate the pathophysiology of thyroid dysfunction induced by COVID 19 at both molecular and clinical levels. Furthermore, future prospective studies are crucial to clarify the prevalence of thyroid function alterations in COVID-19 patients, as well as to provide more clinical data to elucidate how it could impact the disease outcome.

## Author contributions

The attributions the authors had in the production of the manuscript were: Literature review and article writing: CR, JC and FH; Text review and interpretation of data for the work: AF, RF, DC and HR; Figure creation: FH; Data collection: FB, Text review: FB, DC and HR and research coordinator and text review: DC. All authors contributed to the article and approved the submitted version.
